# Co-Variation of Bacterial and Fungal Communities in Different Sorghum Cultivars and Growth Stages is Soil Dependent

**DOI:** 10.1007/s00248-017-1108-6

**Published:** 2017-11-16

**Authors:** Thiago R. Schlemper, Johannes A. van Veen, Eiko E. Kuramae

**Affiliations:** 1 Department of Microbial Ecology, Netherlands Institute of Ecology (NIOO-KNAW), Wageningen, The Netherlands; 2 Department of Biology, Leiden University, Leiden, The Netherlands

**Keywords:** 16S rRNA, 18S rRNA, Rhizosphere, Sorghum bicolor genotypes

## Abstract

Rhizosphere microbial community composition can be influenced by different biotic and abiotic factors. We investigated the composition and co-variation of rhizosphere bacterial and fungal communities from two sorghum genotypes (BRS330 and SRN-39) in three different plant growth stages (emergence of the second leaf, (day10), vegetative to reproductive differentiation point (day 35), and at the last visible emerged leaf (day 50)) in two different soil types, Clue field (CF) and Vredepeel (VD). We observed that either bacterial or fungal community had its composition stronger influenced by soil followed by plant growth stage and cultivar. However, the influence of plant growth stage was higher on fungal community composition than on the bacterial community composition. Furthermore, we showed that sorghum rhizosphere bacterial and fungal communities can affect each other’s composition and structure. The decrease in relative abundance of the fungus genus *Gibberella* over plant growth stages was followed by decrease of the bacterial families *Oxalobacteracea* and *Sphingobacteriacea*. Although cultivar effect was not the major responsible for bacterial and fungal community composition, cultivar SRN-39 showed to promote a stronger co-variance between bacterial and fungal communities.

**Electronic supplementary material:**

The online version of this article (10.1007/s00248-017-1108-6) contains supplementary material, which is available to authorized users.

## Introduction

The rhizosphere harbors a wide range of microorganisms, which have been shown to influence significantly plant growth, root architecture, and nutrient uptake [1–4]. Conversely, the composition of microbial rhizosphere communities is influenced by biotic and abiotic factors including plant species (or genotypes) and soil management [5–7].

Studies on the impact of different soil fertilization managements on the composition of the bacterial community in the rhizosphere of sorghum have shown that the bacterial community is more affected by compost than by inorganic fertilizers [8]. In addition, geographic location and soil characteristics are the main factors explaining the variability in the structure of the bacterial community in the rhizosphere of sorghum [9]. Moreover, in an earlier study, we found soil to be the most important factor on sorghum rhizosphere bacterial community assembly followed by plant growth stage and plant genotype [10]. Furthermore, we found that along plant growth stage, the impact of soil on the bacterial community assembly reduced and, unlike, the impact of plant genotype increased.

Most of rhizosphere community studies focus on either bacterial or fungal communities. However, the dynamics of both combined communities in different plant species are rather uncommon, but are of great relevance. Marschner et al. [11] showed that arbuscular mycorrhizal fungi (AMF) infection changes the bacterial community composition in the rhizosphere of maize with time. While studying the impact of elevated atmospheric CO_2_ on the carbon flow in the rhizosphere in *Festuca rubra*, Drigo et al. [12] found that the allocation of labile photosynthates from AMF to soil promoted shifts on fungal and bacterial rhizosphere microbial communities. Vázquez et al. [13] showed that the interaction between AMF and the microbial inoculants *Azospirillum*, *Pseudomonas*, and *Trichoderma* induced changes in the microbial population in the rhizosphere of maize. Additionally, through the taxonomic assignment of the annotated rRNA and mRNA reads Chapelle et al. [14] found that *Sphingobacteriaceae* and *Oxalobacteraceae* were more abundant in rhizosphere of sugar beet inoculated with *Rhizoctonia solani* than in non-fungal inoculated plant cultivated in suppressive soil. However, these studies are focused in a single group or single species of fungi effect on bacterial community.

Although studies of combined fungal and bacterial diversity and community composition have been performed in rhizosphere, very few studies have directly correlated the composition of one community to another [15, 16]. Particularly in sorghum, as far as we know, there are no studies on mutual effects on the composition and diversity of bacteria and fungi in the rhizosphere. *Sorghum bicolor* (L.) Moench is the fifth cereal most produced worldwide and is a staple food for more than 500 million people in 30 countries [17]. Sorghum is considered to be drought and salinity tolerant and its adaptation to low fertility soils allows the cultivation of this cereal in tropical areas under adverse climate conditions [18]. Here, we aimed to evaluate the variation of fungal and bacterial communities and the relationship of both communities in rhizosphere of different sorghum genotypes in different soils. We tested the hypothesis that (i) fungal-bacterial interaction in the sorghum rhizosphere is modulated by the tripartite factors: plant genotype, soil type, and plant growth stage and (ii) fungal and bacterial rhizosphere communities compositions are modulated by changes in each other’s abundances.

## Material and Methods

### Soil Sampling

The soils were collected from two locations in The Netherlands: Clue field (CF) (52°03′37.91″ N and 5°45′7.074″ E) characterized as Arenosol soil (natural soil on former but abandoned field) and Vredepeel (VD) (51°32′25.8″ N and 5°51′15.1″ E) characterized as Gleyic Podzol soil (agriculture field). From each area, the soil samples were collected (0–20 cm topsoil layer) from five points equidistant at 50 m from each other. Once collected, the soil was sieved (4 mm mesh size) and homogenized. The physical and chemical characteristics of each soil are described in Table S1.

### *Sorghum bicolor* Cultivars and Mesocosm Experiment

Two different cultivars from different origins were chosen to assess the bacterial and fungal communities composition in the rhizosphere of *S. bicolor*: BRS330 cultivar—a hybrid grain resistant to anthracnose, leaf blight, leaf rust, and sooty stripe [19, 20]—and cultivar SRN-39 (grain)—a high producer of orobanchol (strigolactone molecule) root exudate [10] and resistant against the root parasitic weed *Striga hermonthica* (Del.) Benth [21]. The seeds of cultivar BRS330 were from “Embrapa Milho e Sorgo” (Brazil) and the seeds of cultivar SRN-39 originally released in Niger and Sudan (Africa) [22, 23] were provided by the Laboratory of Plant Physiology—Wageningen University (Netherlands).

The experimental design and sampling consisted of three replicates of two soil types, two sorghum cultivars, and three plant growth stages, in total 36 experimental units randomly distributed in a greenhouse. Fifteen seeds of each sorghum cultivar were sown in soils in plastic pots (6.5 L). The pots were kept under controlled temperature and photoperiod conditions (22 °C/17 °C day/night and photoperiod 16/8 h light/dark). After 5 days, plantlets were trimmed to five seedlings per pot. Rhizosphere soil was sampled after in three different plant growth stages: at the emergence of the second leaf (day10), at the emergency of the fifth leaf when the plants migrate from vegetative to reproductive differentiation point (day 35), and at the last visible emerged leaf (day 50) before the plant flowering. At the first stage of plant growth (day 10), rhizosphere soil was sampled removing the whole plant and brushing the soil adhered to the seminal roots, and for the last stages of plant growth (days 35 and 50), rhizosphere soil was sampled with a cylindrical auger (6 × 150 mm). Bulk soil samples were taken from pots without plants. Rhizosphere and bulk soil samples for DNA extraction were kept at − 80 °C.

### DNA Extraction and 16S rRNA Partial Gene Sequencing

DNA was extracted from 0.25 g of soil of each sample using DNA Power soil DNA isolation kit (Mo Bio Laboratories, Inc., Carlsbad, CA, USA). DNA integrity was checked by agarose gel (1.5%) electrophoresis in TBE (Tris-borate-EDTA) buffer. DNA from each treatment was used as template for 16S rRNA and 18S rRNA partial genes fragments amplification. The amplification of the 16S rRNA partial gene was performed using the primer set 515F and 806R [24]. Primers contained multiplex tags for sample identification. PCR was carried out using 0.2 μl of 0.056 U fast StartExp*Taq* Polymerase (Roche Applied Sciences, Indianapolis, IN, USA), 2.5 μl dNTP (2 mM each), 0.25 μl of each primer, and 1.0 μl of DNA template. Thermocycling conditions were as follows: denaturing at 95 °C for 5 min followed by 35 cycles of denaturation at 95 °C for 30 s, annealing at 53 °C for 30 s, extension at 72 °C for 60 s followed by a final extension at 72 °C for 10 min. As negative control, water was used instead of DNA, and as positive control DNA of *Escherichia coli* was used. For the 18S rRNA partial gene amplification, a fungal-specific primer set FR1 and FF390.1 [25] was used to amplify a 350 bp region of the 18S rRNA gene. Primers contained multiplex tags for sample identification. PCR reactions were carried out using 2.5 μl of 2 mM dNTP, 0.5 μl of each primer, 1.0 μl of DNA template, and 0.2 μl of 0.056 U of Fast StartExp-Polymerase (Roche Applied Sciences, Indianapolis, IN, USA). The PCR reaction had an initial denaturation at 95 °C for 5 min, followed by 35 cycles of denaturation at 95 °C for 30 s, annealing at 58 °C for 30 s, extension at 72 °C for 60 s, and the final extension at 72 °C for 10 min. As negative control, water was used instead of DNA. The PCR products were purified using QIAquick PCR Purification Kit (Qiagen Technologies) and their quality were checked before and after the purification in agarose gel electrophoresis in TBE buffer. The PCR amplicons were quantified using Fragment analyser™—Automated CE system (Advanced Analytical Technologies, Inc) and equimolar pooled. The samples were sequenced in PGM machine on Ion Torrent (Life technology) in Korea (Macrogen Inc. Company, South Korea).

## Data Analyses

### 16S and 18S rRNA Sequences Processing

Forward and reverse primer sequences in the library FASTQ file of each sample were removed using Flexbar version 2.5 [26]. Sequences were filtered for quality criteria with a Phred quality score of 25 and with minimum sequence length of 150 bp by running the FASTQ-MCF [27]. After filtering, FASTQ files were converted to FASTA format and concatenated into a single file. Chimera sequences were detected using the UCHIME algorithm implemented in VSEARCH [28]. The reads were clustered into Operational Taxonomic Units (OTU), within evolutionary distance of 97% using the UPARSE [29] performed with VSEARCH version 1.0.10 [30]. The OTU table was converted to biological observation matrix (BIOM) format 1.3.1 [31] and using the RDP Classifier version 2.10 [32], taxonomic information for each OTU was added to the BIOM file. All procedures were implemented in a Snakemake workflow [33]. The number of sequences in each library was rarefied (*alpha_rarefaction.py*) to 2.000 sequences for bacteria and to 550 sequences for fungi prior to diversity analyses in QIIME 1.8.0 [34]. The 16S rRNA and 18S rRNA sequence data are available at the European Nucleotide Archive (ENA) (https://www.ebi.ac.uk/ena/) under the study accession number PRJEB21895 (ERP024198).

### Statistical Analyses

To check the treatment effects on sorghum rhizosphere bacterial and fungal communities composition, between-classes analysis (BCA) and co-inertia analysis (COIA) were performed in R v3.3.3 using the package “ade4” [35]. To explore the dissimilarities of treatments within each community, a principal component analysis (PCA) was used to create BCA tables using the function “bca.” In order to find the similarity of bacterial and fungal community within treatments, BCA tables were used to conduct co-inertia analysis for the two soils using the function “coinertia.” Monte-Carlo test was applied for BCA and COIA using 999 random permutations. For co-inertia, “RV.test” R function was used to perform Monte-Carlo test. As a result of COIA, plots with arrows are formed. The back of the arrow represents the location of bacterial community organisms and the tip of arrow represents the location of fungal community organisms. The strength of the relationship between both communities is inversely related to the length of the arrow. Arrows projected to the same direction showed strong association between the treatments with respect to the microbial composition [36]. Bacterial and fungal community structure co-variance scores were given by COIA analysis. Family groups responsible for such co-variance were those had higher score than the 95% of sample normal distribution. This was calculated by the standard deviation multiplied by 1.96, what is the range that corresponds to 95% of normal distribution of the standard deviation.

To infer how the rhizosphere bacterial community co-varied with the factors soil, cultivar, and plant growth stage, the bacterial and fungal abundance data were transformed by Hellinger transformation [37] using the package Vegan version 2.4.0 [38] and the co-variance was measured by the coefficient RV-Value by multiple factor analysis (MFA) using the package “FactoMineR” [39] in R v3.1.3 program. Moreover, using the same R package, we applied permutational multivariate analysis of variance (PERMANOVA) using Bray-Curtis distance matrix with 999 permutations to test the influence of the factors soil, plant growth stage, and cultivar in the rhizosphere bacterial and fungal community.

In order to check for dissimilarities within the microbial communities, treatments were divided into subsets and principal coordinate analysis (PCoA) were performed in QIIME 1.9.1 using the script *beta_diversity_through_plots.py* with Bray-Curtis distance matrices. Distance matrices generated by PCoA were used to perform PERMANOVA analysis with 9999 random permutations (*P* < 0.05). For the PCoAs where the treatment effects were significant, microbial community family groups responsible for the dissimilarities were checked. Differences in mean proportion was tested through Welch’s test (*P* < 0.05) using the Statistical Analysis of Metagenomics Profiles (STAMP) v2.1.3 program [40]. To avoid false discovery rates (FDR), Benjamini-Hochberg [41] was applied.

Alpha diversity index (Shannon), species richness (Chao1), as well as the total number of OTUs were calculated in QIIME 1.9.1 using the command *alpha_diversity.py.* In order to check for significant differences among samples, analysis of variance ANOVA and Tukey test (*P* < 0.05) was performed in R for each Alpha diversity index.

## Results

Analysis of co-inertia (RV-coefficient) at family level revealed that soil type, plant growth stage, and cultivar explained 52.62, 22.70, and 12.73% of the rhizosphere bacterial community variation, respectively (Table S2). For the fungal community, soil type, plant growth stage, and cultivars explained 42.83, 26.02, and 14.99%, of the variation, respectively (Table S3). We tested the statistical significance of the factors soil, plant growth stage, and cultivar on the rhizosphere bacterial and fungal community structures by PERMANOVA using Bray-Curtis as distance matrix. The results showed that soil had significant effects on both the bacterial (*F* = 6.87; *P* < 0.001) and fungal (*F* = 7.89; *P* < 0.001) communities; plant growth stage had a significant effect only on the fungal community (*F* = 2.68; *P* < 0.001) and cultivar had no significant effect on both communities (Table S4).

### Differences in Bacterial Community Structure

**Fig. 1:**
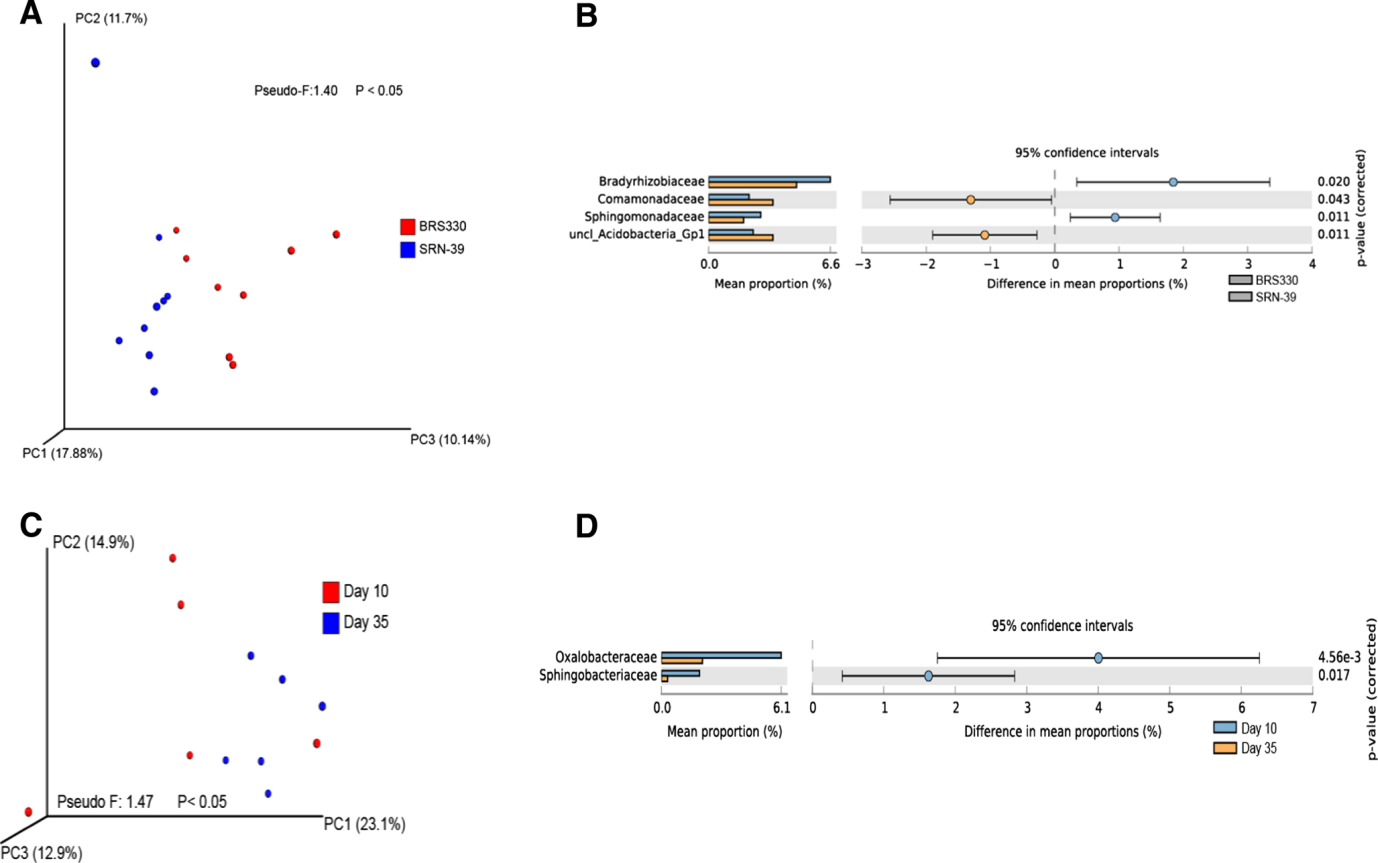
**a** Principal coordinate analysis (PCoA) and **b** differences in relative abundance of bacteria families between cultivars BRS330 and SRN-39, and **c** PCoA and **d** differences in relative abundance of bacteria families between days 10 and 35 at Clue field soil (Welch’s test; *P* < 0.05)

PERMANOVA test showed that the bacterial communities from the bulk soils of CF and VD were not significantly different (Pseudo-*F*: 1.40; *P* = 0.40) (Fig. S1). However, the same analysis, showed that the bacterial community was significant different in the rhizosphere soils of CF and VD (Pseudo-*F*: 6.9; *P* < 0.05) (Fig. S2). Through Welch’s test, we found that among the bacteria families driving this dissimilarity, *Bradyrhizobiaceae* was more abundant in rhizosphere soil of CF than VD, whereas *Caulobactereaceae*, *Phyllobacteriaceae*, and *Xanthomonadaceae* were more abundant in VD (Fig. S2). Welch’s test revealed a significant difference in rhizosphere bacterial composition between both CF (Pseudo-*F*: 2.3; *P* < 0.05) and VD (Pseudo-*F*: 2.55; *P* < 0.05) soils (Fig. S3). At CF soil, this difference was mainly caused by unclassified *Spartobacteria* family with high abundance in bulk soil, and *Comamonadaceae*, *Oxalobacteraceae* families and unclassified Alphaproteobacteria with higher abundances in the rhizosphere than in the bulk soil (Fig. S3). At VD soil, *Oxalobacteraceae* as well as organisms that could not be classified at family taxonomic level belonging to *Acidobacteria* Gp1, *Myxococcales* (Gammaproteobacteria), and Proteobacteria were significantly more abundant in rhizosphere than in bulk soil (Welch’s test; *P* < 0.05) (Fig. S3). PERMANOVA analysis comparing cultivars in the CF soil showed that the rhizosphere bacterial community of cultivar BRS330 significantly differed from that of cultivar SRN-39 (Pseudo-*F*: 1.14; *P* < 0.05) (Fig. 1a). Performing Welch’s test we found *Bradyrhizobiaceae* and *Sphingomonadaceae* with mean proportion significant highest in rhizosphere of BRS330, whereas *Comamonadaceae* and unclassified *Acidobacteria* Gp1 were significant highest in SRN-39 rhizosphere (*P* < 0.05) (Fig. 1b).

The only significant difference in the bacterial community composition found over growth stages was in CF soil between the day 10 and 35 of plant growth (Pseudo-*F*: 1.47; *P* < 0.05) (Fig. 1c). The two families responsible for this dissimilarity were *Oxalobacteraceae* and *Sphingobacteriaceae* with significant highest abundance at day 10 and not at day 35 (*P* < 0.05) (Fig. 1d).

### Differences in Fungal Community Structure

**Fig. 2:**
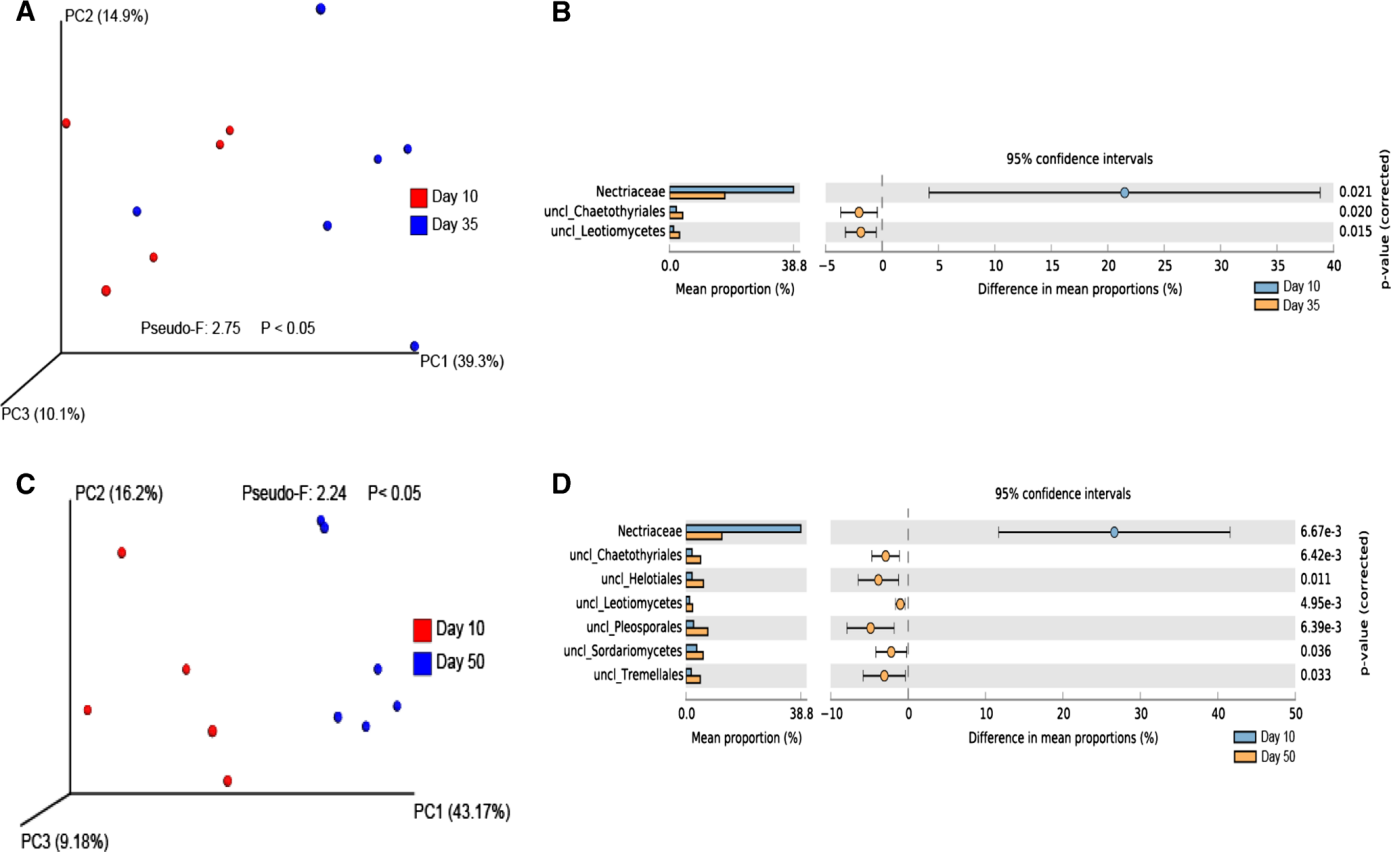
**a** Principal coordinate analysis (PCoA) and **b** differences in relative abundance of fungi families between days 10 and 35, and **c** PCoA and **d** differences in relative abundance of fungi families between days 10 and 50 in Clue field rhizosphere samples (Welch’s test; *P* < 0.05)

The fungal community in both CF and VD bulk soils did not significantly differ (Pseudo-*F*: 2.00; *P* = 0.20) (Fig. S4). However, the fungal rhizosphere community in CF soil was significantly different from that in VD soil (Pseudo-*F*: 7.9; *P* < 0.05) (Fig. S5). *Hypocreaceae* and unclassified *Mortierellales* were more abundant in the sorghum rhizosphere in CF soil than in VD soil. In contrast, the organisms that could not be classified at the family level belonging to the groups of *Saccharomycetales*, *Sordariales*, and *Sordariomycetes* were significantly more abundant in the rhizosphere community in VD than in CF soil (Fig. S5). PCoA showed a clear distinction in the rhizosphere fungal communities at day 10 as compared to day 35 (Pseudo-*F*: 2.75; *P* < 0.05) and 50 (Pseudo-*F*: 2.24; *P* < 0.05) in CF soil (Fig. 2a, c). *Nectriaceae* was found to be the major group responsible for these dissimilarities with higher abundance at day 10 than at days 35 and 50. On the other hand, the abundances of unclassified *Chaetothyriales* and unclassified *Leotiomycetes* were lower at day 10 than at days 35 and 50 (Fig. 2b, d). Overall, *Nectriaceae* was the most abundant fungal family in the Clue field rhizosphere soil (Fig. S6).

**Fig. 3:**
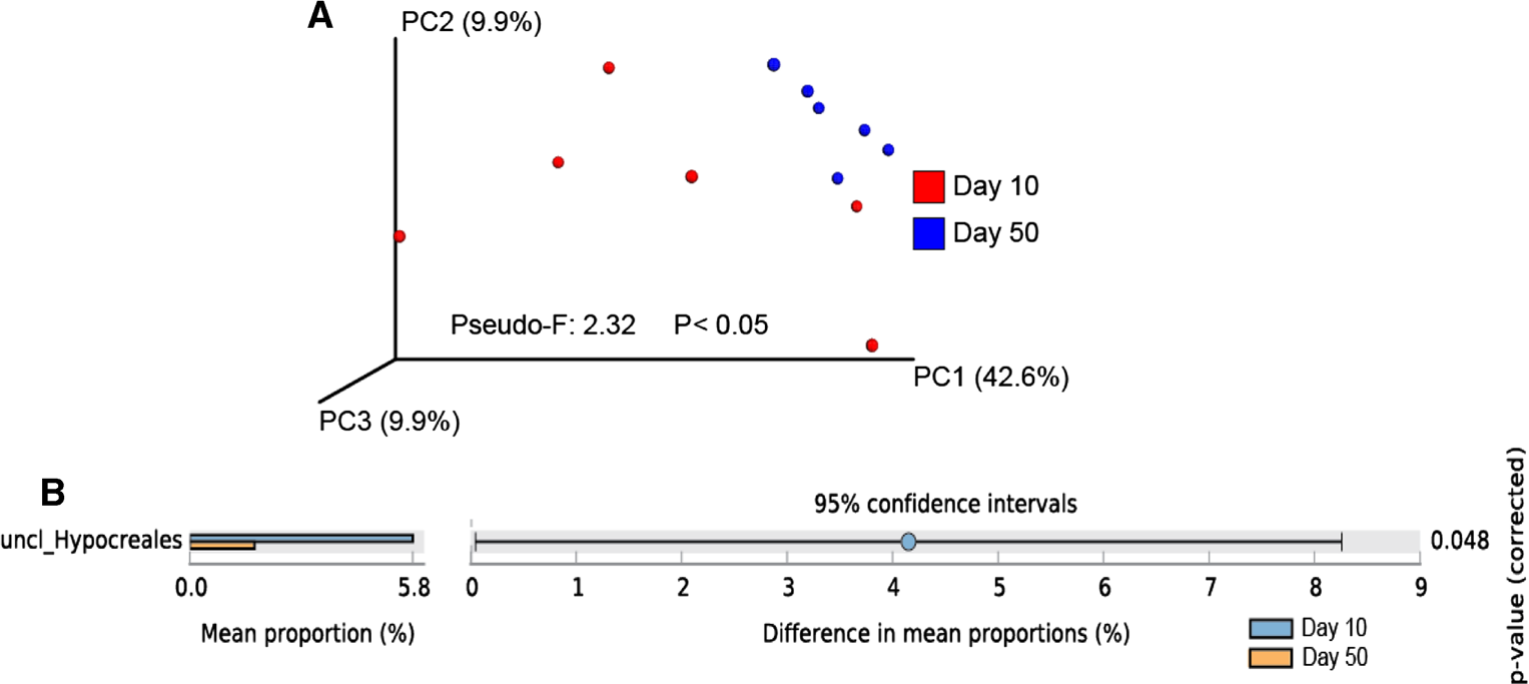
**a** Principal coordinate analysis (PCoA) and **b** differences in relative abundance of fungi families between days 10 and 50 in Vredepeel rhizosphere samples (Welch’s test; *P* < 0.05)

In VD soil, the rhizosphere fungal community also showed to be different between early (day 10) and late (day 50) plant growth stages (Fig. 3a). Despite the difference in rhizosphere fungal community presented by PCoA plot and PERMANOVA analysis, only one fungal group could be assigned to be responsible for this dissimilarity; unclassified *Hypocreales* showed higher abundance at day 10 than at day 50 of plant growth (Fig. 3b).

### Between-Class and Co-Inertia Analyses

**Fig. 4:**
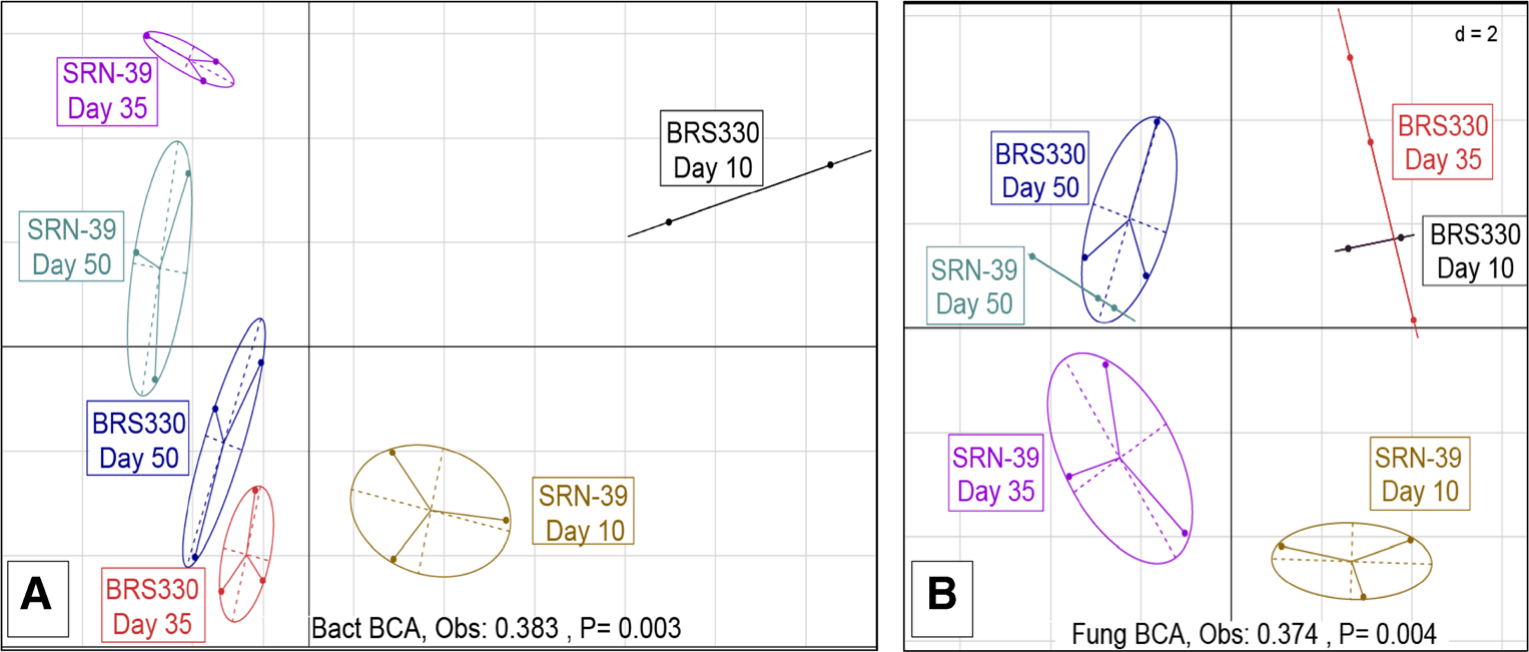
Between-class analysis (BCA) of **a** bacterial and **b** fungal communities in sorghum rhizosphere of cultivars BRS 330 and SRN-39 at days 10, 35, and 50 of plant growth stage in Clue field soil

**Fig. 5:**
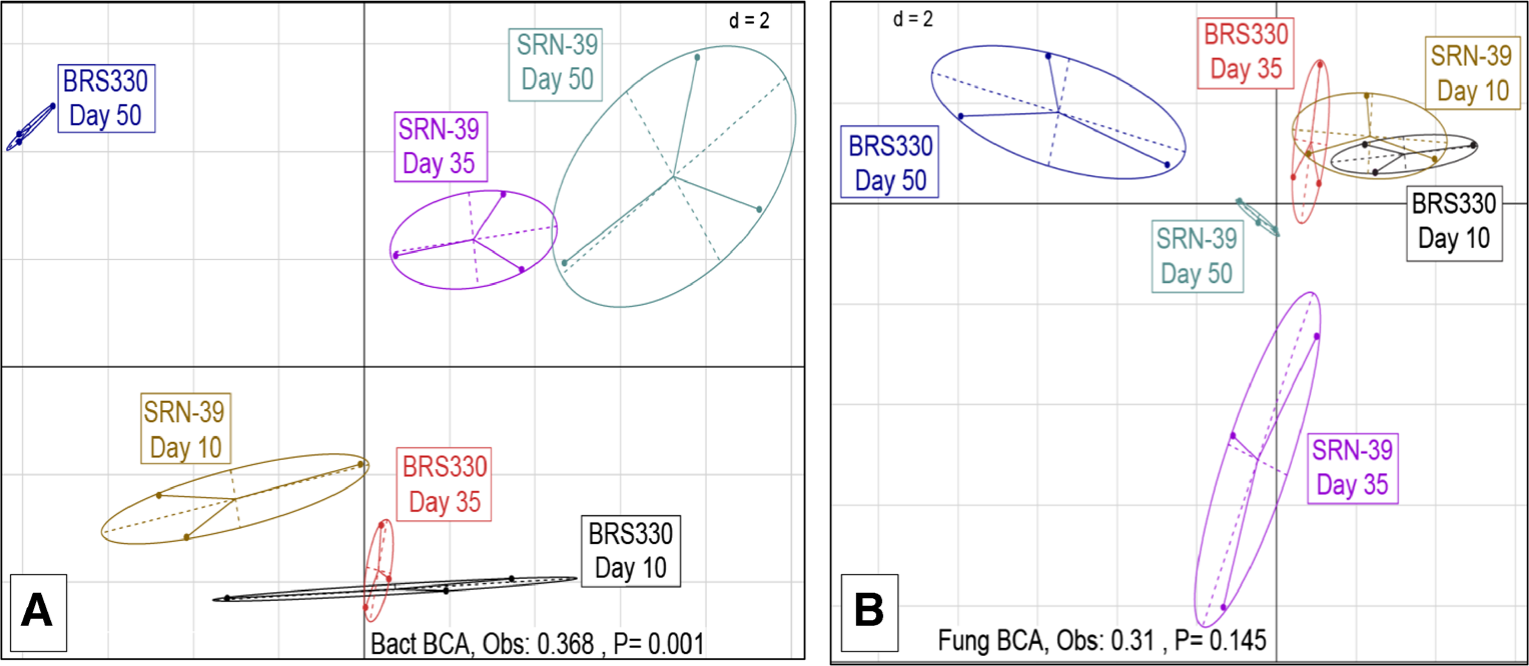
Between-class analysis (BCA) of **a** bacterial and **b** fungal communities in sorghum rhizosphere of cultivars BRS 330 and SRN-39 at days 10, 35, and 50 of plant growth stage in Vredepeel soil

Between classes analysis (BCA) was performed to check for dissimilarities in the total microbial rhizosphere community. At CF soil, bacterial and fungal communities composition were significantly different across sorghum treatments explaining 38% (*P* = 0.03) and 37% (*P* = 0.04) of total variation, respectively. Ellipses representing bacterial community composition of the cultivars BRS330 and SRN-39 at early sampling showed a clear separation from the ellipses of the two later samplings (Fig. 4a). For the fungal community, although this separation remained consistent for cultivar SRN-39, for cultivar BRS330, the ellipse separation was more evident in the last sampling (day 50) than the early sampling points (days 10 and 35) (Fig. 4b). At VD soil the rhizosphere bacterial community composition was significantly different among sorghum treatments, explaining 36.8% (*P* = 0.001) of the total variation. Ellipses dispositions representing the bacterial community of cultivar BRS330 showed a clear separation between the composition of days 10 and 35 to the day 50 of plant growth. Conversely, bacterial community present in rhizosphere cultivar SRN-39 showed similarity between the latest two stages of plant growth (days 35 and 50) with dissimilarity to the day 10 of plant growth (Fig. 5a). No significant difference was found for rhizosphere fungal community at VD soil (Monte-Carlo test) (Fig. 5b).

**Fig. 6:**
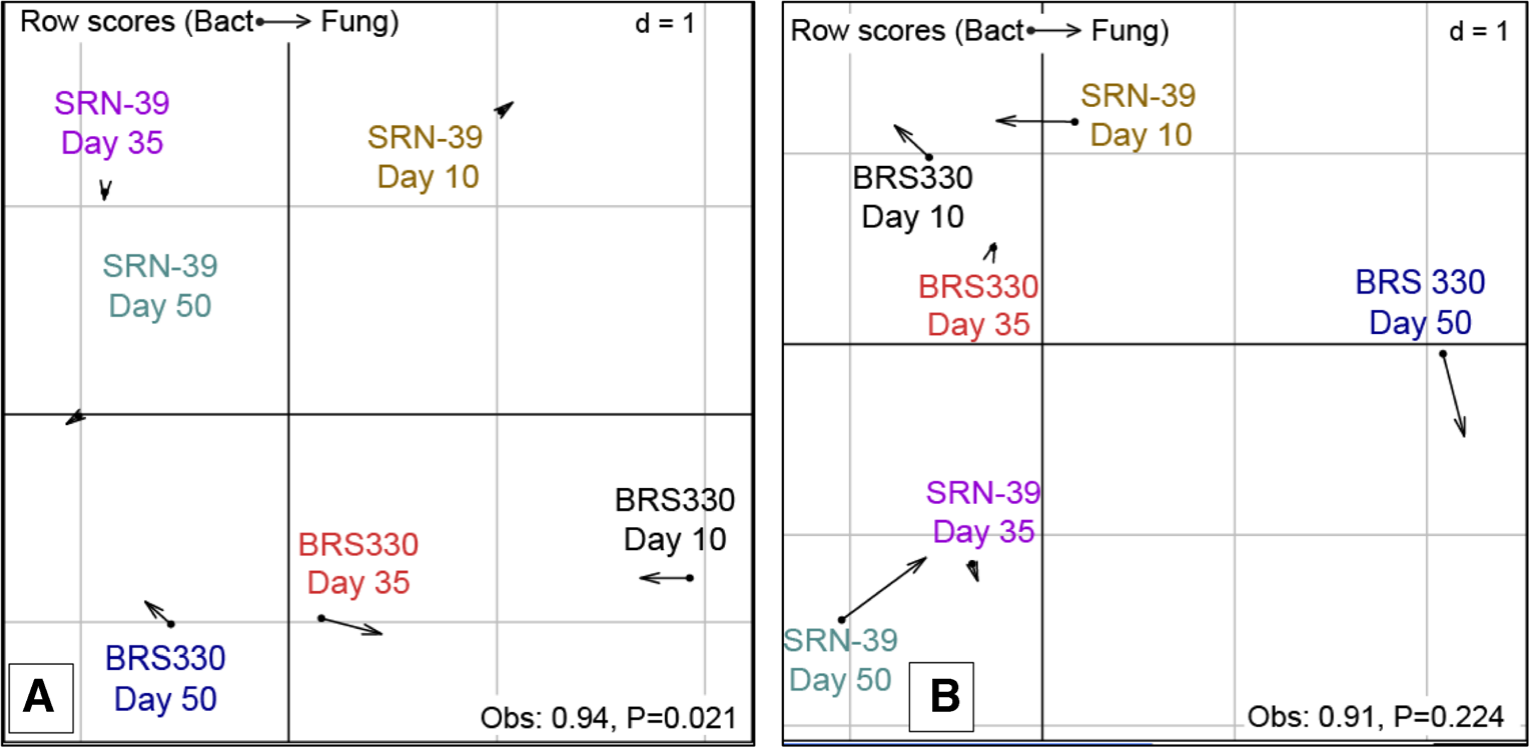
Co-inertia analysis (COIA) of bacterial and fungal communities in **a** Clue field and **b** Vredepeel soils. Arrows represent the co-variation of both communities within the treatments: cultivar BRS 330 and SRN-39 at days 10, 35, and 50 of plant growth stage

Co-variance between rhizosphere bacterial and fungal community structures was determined using co-inertia analysis (COIA). Plotting bacterial and fungal community’s ordination together resulted in a new ordination plot where an arrow links bacterial to fungal community positions. We observed that treatments in CF and VD soils explained 94 and 91% of the rhizosphere microbial community variation, respectively (Fig. 6a, b). The variation between the bacterial and fungal communities was significantly different in CF soil (*P* = 0.02). Shorter arrows in cultivar SRN-39 than in cultivar BRS330, in each growth stage, indicate stronger relationship between bacterial and fungal communities in the SRN-39 rhizosphere than in the BRS330 rhizosphere. For cultivar SRN-39, the projection of arrows by day 10 in the opposite direction of days 35 and 50 of plant growth showed that day 10 had a weak similarity on the variation of bacterial-fungal communities compared with days 35 and 50 of plant growth stage. No significant difference was found for VD soil (*P* = 0.22) (Monte-Carlo test) (Fig. 6b). For each soil, we assessed the representatives of rhizosphere bacterial and fungal communities responsible for the co-variance of each co-inertia axis (Tables S5 and S6).

### Alpha Diversity

For bacteria community, Tukey tests applied to all alpha diversity indices (number of OTUs, Chao1 and Shannon (H′)) showed no significant differences between VD and CF bulk soils (*P* > 0.05). The rhizosphere bacterial community of cultivar SRN-39 at day 10 had significant lower number of OTUs and lower diversity (Shannon H′) in CF than in VD soil. No significant difference in Shannon diversity, Chao1 or number of OTUs was found comparing bulk soil and rhizosphere in CF soil. The rhizosphere community of both cultivars grown in VD soil, at each growth stage, showed higher bacterial diversity and number of OTUs than in bulk soil. However, for both cultivars planted in VD soil, no difference was found among the OTUs and diversity of rhizosphere bacterial community throughout sampling time. For both cultivars planted in CF soil, the richness of the rhizosphere bacterial community was not different from that of bulk soil. In VD soil, the richness (Chao1) in the rhizospheres of cultivars BRS330, at day 10 and SRN-39 at day 50 was significantly higher than the bulk soil, whereas no significant difference was evidenced among rhizosphere treatments (Table S7). For fungal community, no difference in alpha diversity was found (Table S8).

## Discussion

Our first hypothesis that fungal-bacterial interaction in the sorghum rhizosphere is modulated by the tripartite factors: plant genotype, soil type, and plant growth stage is accepted. Out results showed that for both bacterial and fungal communities, soil plays the major role in their assembly in sorghum rhizosphere. Although bacterial and fungal community structures showed the same trend regarding to the influence of soil, growth stage, and sorghum cultivar, fungal communities showed to be more influenced by plant growth stage than bacterial communities. Similarly, Han et al. [42] found plant growth stage a dominant factor determining the structure of the fungal community as compared to edaphic factors in the soybean rhizosphere. We suggest that the fungal community composition was more affected by plant growth stage than the bacterial community composition as the result of the versatility that fungi can interact with plants in different stages of plant development, acting as pathogens, symbionts, and saprotrophic [43–45]. Moreover, plants release different exudates of different chemical structure complexities during different growth stages [46], which may have larger effects on fungi in the rhizosphere than on bacteria.

The influence of plant growth stage on the fungal rhizosphere community is evidenced by the significant higher relative abundance of *Nectriaceae* at day 10 (38.8%) compared with day 35 (18%) and 50 (12%) in the CF soil. *Nectriaceae* showed the highest relative abundance (21%) among fungal families, all belonging to the *Gibberella* genus (Fig. S6). Similar results were found by Grudzinska-Sterno et al. [47] in wheat growth stages that *Gibberella avenacea* significantly decreased, at least four times fold, from young to mature plants. All *Gibberella* species are sexual stages of *Fusarium* species [48], which genus contains many plant pathogens and mycotoxin producers, being of great agricultural and economical importance [49]. At CF soil, the bacterial families of *Sphingobacteriaceae* and *Oxalobacteraceae* decreased significantly in time. Corroborating with our findings, Green et al. [50] studying the bacterial community composition of cucumber root observed a decrease in abundance of *Oxalobacteraceae* from early to late plant growth stage. The second hypothesis that fungal and bacterial rhizosphere communities’ compositions are modulated by changes in each other’s abundances is accepted. Although the relationship in the observed abundances of *Sphingobacteriaceae* and *Oxalobacteraceae* bacteria and *Gibberella* fungi was not experimentally assessed, we suggest that there may be some link between these organisms, as both bacterial families are known to be antagonist to fungal activity. *Oxalobacteraceae* were reported to have antifungal, chitinolytic, and mycophagous characteristics, being suppressive toward fungi plant pathogens including *Fusarium* species [44, 51, 52]. Moreover, *Fusarium* species are known to produce oxalic acid [53] that may have attracted members of *Oxalobacteraceae* that are characterized for their ability to degrade oxalate [54, 55].

Although the effect of plant growth on the dissimilarity of fungal community was evidenced for both soils, this effect was stronger in CF than VD soil. Furthermore, bacterial and fungal communities showed significant variation between each other at CF soil, whereas no difference was verified in VD soil. We hypothesize that influence of CF soil on microbial community variation is linked with low soil fertility. The fertility of CF measured by the sum of bases was less than half of that of VD soil [10]. Additionally, at CF soil the co-variance of bacterial and fungal communities of the rhizosphere of cultivar SRN-39 was higher than at cultivar BRS330 for all plant growth stages. Although cultivar had smaller effects on the selection of bacterial and fungal communities, it may play an important role in the interaction of both microbial communities. However, given the relative small effects of cultivars and growth stages on rhizosphere microbial community composition, we conclude that the effects of growth stage and cultivar differences on microbial community composition were soil dependent.

The initial community (bulk soil) either for bacterial or fungal community did not differ between both soils regarding α and β-diversity. However, soils showed to have different microbial community β-diversity composition at the rhizosphere compartment. We speculate that this difference may be linked with the variation on carbon inputs released by plants to the rhizosphere depending on soil characteristics [56, 57]. The fungal diversity did not differ among treatments for the both soils.

The results revealed in this work lead us to the conclusion that fungal and bacterial communities varied with each other in sorghum rhizosphere. The strength of this co-variance is dependent of soil, plant growth stage, plant genotype, and microbial composition. Although cultivar effect was not the major responsible for bacterial and fungal community composition, cultivar SRN-39 showed to promote a stronger co-variation between bacterial and fungal communities.

## Electronic Supplementary Material

ESM 1 (DOCX 214 kb)

ESM 2 (DOCX 1.8 mb)

ESM 3 (DOCX 1.49 mb)

ESM 4 (DOCX 213 kb)

ESM 5 (DOCX 610 kb)

ESM 6 (DOCX 278 kb)

ESM 7 (DOCX 18.1 kb)

ESM 8 (DOCX 16.6 kb)

ESM 9 (DOCX 16.7 kb)

ESM 10 (DOCX 17.9 kb)

ESM 11 (DOCX 18 kb)

ESM 12 (DOCX 19.1 kb)

ESM 13 (DOCX 20.3 kb)

ESM 14 (DOCX 20 kb)
