# Supplementary material for: Co-Variation of Bacterial and Fungal Communities in Different Sorghum Cultivars and Growth Stages is Soil Dependent
Source: Microb Ecol. 2017 Nov 16;76(1):205–14. doi: 10.1007/s00248-017-1108-6 (PMC6061463; doi:10.1007/s00248-017-1108-6)
Supplement: Supplementary file 4 — (DOCX 213 kb) [file 248_2017_1108_MOESM4_ESM.docx]

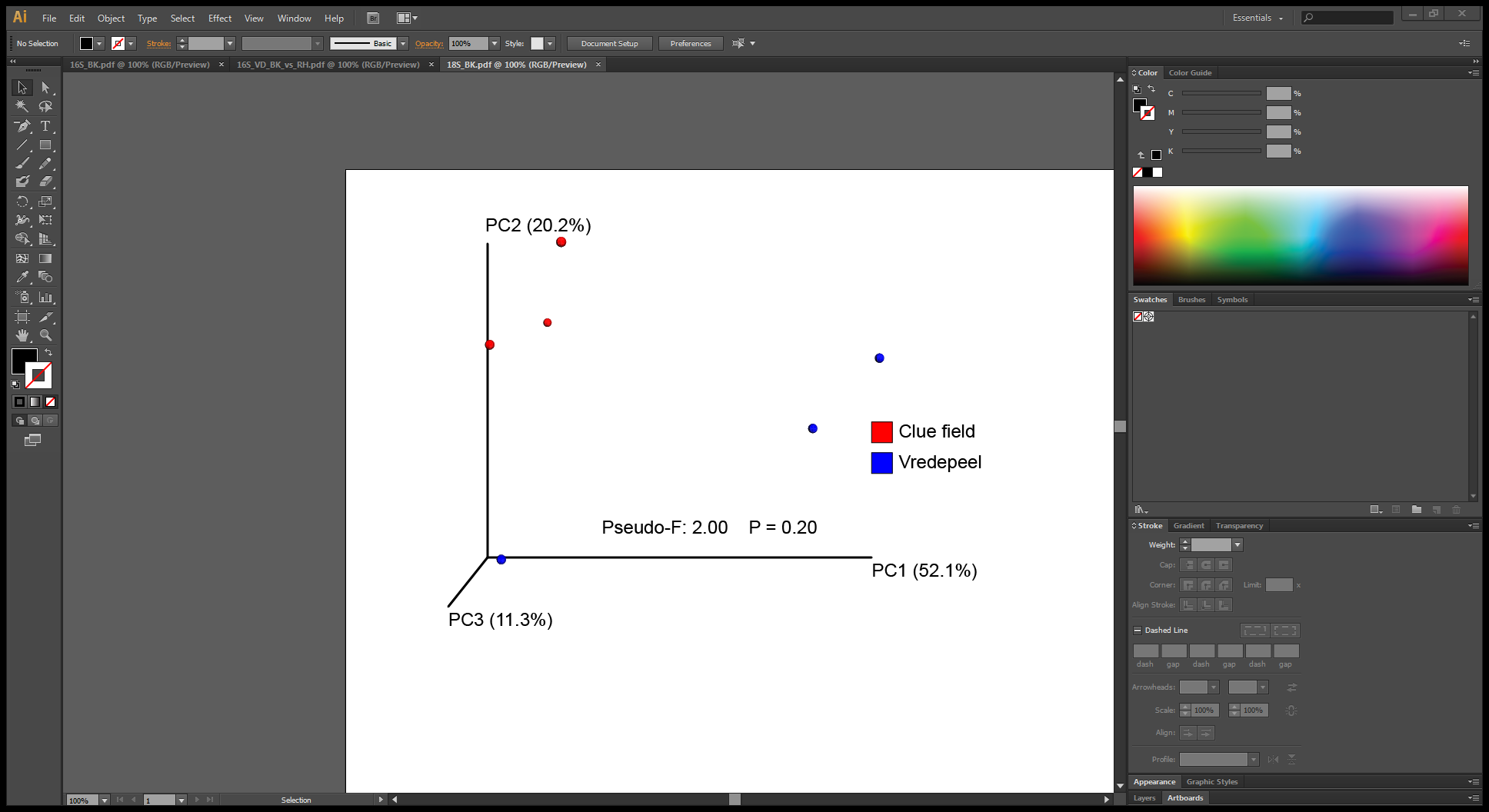


**Fig. S4.** Principal Coordinate Analysis (PCoA) representing the dissimilarity between fungal community present on bulk soil of clue field and vredepeel soils.
