# Supplementary material for: Co-Variation of Bacterial and Fungal Communities in Different Sorghum Cultivars and Growth Stages is Soil Dependent
Source: Microb Ecol. 2017 Nov 16;76(1):205–14. doi: 10.1007/s00248-017-1108-6 (PMC6061463; doi:10.1007/s00248-017-1108-6)
Supplement: Supplementary file 5 — (DOCX 610 kb) [file 248_2017_1108_MOESM5_ESM.docx]

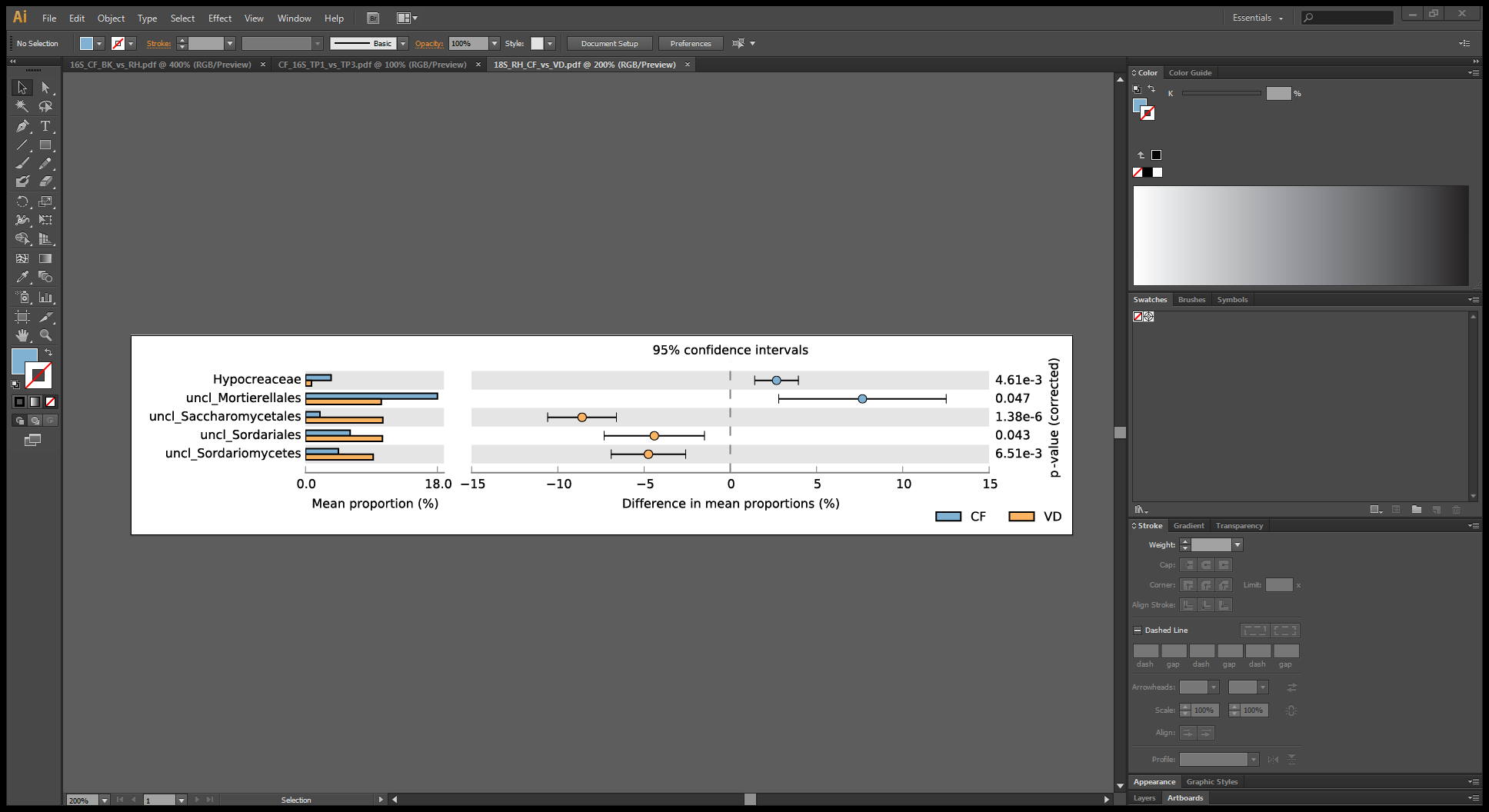

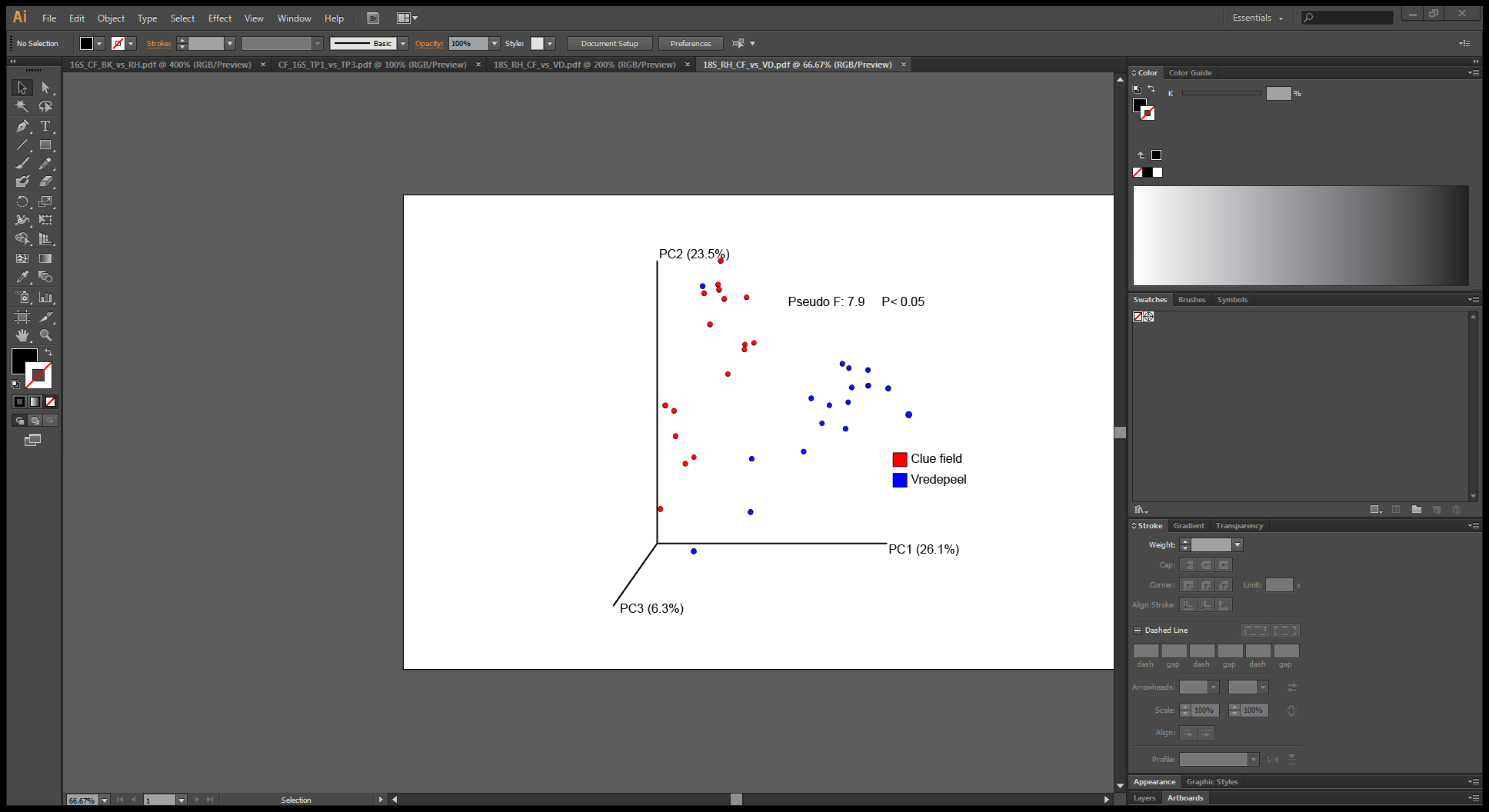


A

B

**Fig. S5.** (A) Principal Coordinate Analysis (PCoA) and (B) differences in relative abundance of fungi families between Clue field and Vredepeel rhizosphere samples (Welch’s test; P<0.05).
