# Supplementary figures and images for: Co-Variation of Bacterial and Fungal Communities in Different Sorghum Cultivars and Growth Stages is Soil Dependent
Source: Microb Ecol. 2017 Nov 16;76(1):205–14. doi: 10.1007/s00248-017-1108-6 (PMC6061463; doi:10.1007/s00248-017-1108-6)

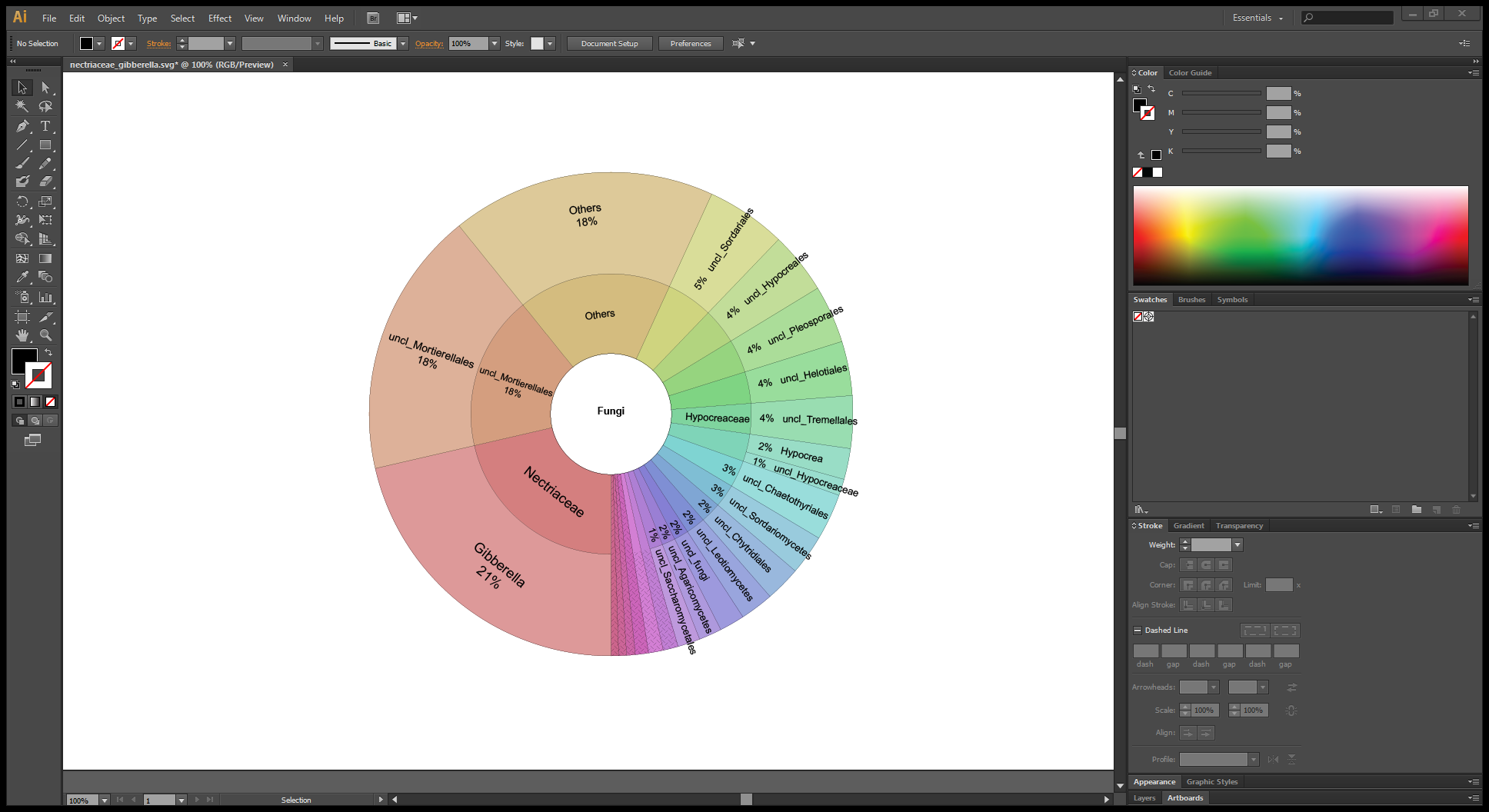


**Fig. S6.** Relative abundance of rhizosphere fungal community in Clue field soil.

Supplement: Supplementary file 6 — (DOCX 278 kb) [file 248_2017_1108_MOESM6_ESM.docx]
