# Supplementary material for: Co-Variation of Bacterial and Fungal Communities in Different Sorghum Cultivars and Growth Stages is Soil Dependent
Source: Microb Ecol. 2017 Nov 16;76(1):205–14. doi: 10.1007/s00248-017-1108-6 (PMC6061463; doi:10.1007/s00248-017-1108-6)
Supplement: Supplementary file 7 — (DOCX 18.1 kb) [file 248_2017_1108_MOESM7_ESM.docx]

**Table S1.** Soil physical and chemical properties of Clue Field and Vredepeel *extracted from Schlemper et al. [10].

| Parameter |  | Unit |  | Soils | | |
| --- | --- | --- | --- | --- | --- | --- |
|  |  |  |  | Clue Field |  | Vredepeel |
| N total |  | mg/Kg |  | 1220 |  | 970 |
| C : N ratio |  |  |  | 18 |  | 22 |
| N supply capacity |  | Kg/ha |  | 43 |  | 24 |
| S total |  | mg/Kg |  | 240 |  | 190 |
| P |  | mg/Kg |  | 5.4 |  | 4.6 |
| K |  | mg/Kg |  | 18 |  | 209 |
| Ca |  | Kg/ha |  | 107 |  | 188 |
| Mg |  | mg/Kg |  | 43 |  | 108 |
| Na |  | mg/Kg |  | 6 |  | 26 |
| pH |  |  |  | 5.1 |  | 5.4 |
| OM |  | % |  | 3.7 |  | 3.7 |
| C inorganic |  | % |  | 0.03 |  | 0.06 |
| SB |  | Cmolc/dm^3^ | | 0.42 |  | 1.53 |
| V |  | % |  | 9.3 |  | 25.6 |
| Clay |  | % |  | 3 |  | 1 |
| Silt |  | % |  | 4 |  | 5 |
| Sand |  | % |  | 89 |  | 90 |
| CEC |  | mmol+/Kg | | 46 |  | 60 |

P, K, Ca, Mg, Na = available; OM= Organic matter; SB: Sum of bases; V: Base saturation; CEC = Cation exchange capacity; N supply capacity = N expected to be mineralized based on N-total, C/N ratio and soil life.
