# Supplementary material for: Co-Variation of Bacterial and Fungal Communities in Different Sorghum Cultivars and Growth Stages is Soil Dependent
Source: Microb Ecol. 2017 Nov 16;76(1):205–14. doi: 10.1007/s00248-017-1108-6 (PMC6061463; doi:10.1007/s00248-017-1108-6)
Supplement: Supplementary file 9 — (DOCX 16.7 kb) [file 248_2017_1108_MOESM9_ESM.docx]

**Table S3.** Inertia co-variance between the factors soil type, growth stage and cultivar with the rhizosphere fungal community

|  | Soil type | | Growth stage | | Cultivar | | Fungi | | |
| --- | --- | --- | --- | --- | --- | --- | --- | --- | --- |
| Soil type | | 100.00% | |  | |  | |  |  |
| Growth stage | | 0.00% | | 100.00% | |  | |  |  |
| Cultivar | | 0.00% | | 0.00% | | 100.00% | |  |  |
| Fungi | | **42.83%** | | **26.02%** | | **14.99%** | | 100.00% |  |
