# Supplementary material for: Co-Variation of Bacterial and Fungal Communities in Different Sorghum Cultivars and Growth Stages is Soil Dependent
Source: Microb Ecol. 2017 Nov 16;76(1):205–14. doi: 10.1007/s00248-017-1108-6 (PMC6061463; doi:10.1007/s00248-017-1108-6)
Supplement: Supplementary file 10 — (DOCX 17.9 kb) [file 248_2017_1108_MOESM10_ESM.docx]

**Table S4.** Permutational Multivariate Analysis of Variance (Adonis) using Bray-Curtis distance matrix for testing the factors soil, plant growth stage and cultivar in rhizosphere bacterial and fungal community

| **Organism** |  | **Factor** |  | **Df** |  | **Sum of Squares** |  | **Mean of Squares** |  | **F** |  | **R2** |  | **P** |
| --- | --- | --- | --- | --- | --- | --- | --- | --- | --- | --- | --- | --- | --- | --- |
| Bacteria |  | Soil |  | **1** |  | **1.16** |  | **1.16** |  | **6.87** |  | **0.17** |  | **0.001** |
|  |  | Growth stage |  | 2 |  | 0.45 |  | 0.23 |  | 1.15 |  | 0.07 |  | 0.18 |
|  |  | Cultivar |  | 1 |  | 0.23 |  | 0.23 |  | 1.16 |  | 0.03 |  | 0.197 |
|  |  |  |  |  |  |  |  |  |  |  |  |  |  |  |
| Fungi |  | Soil |  | **1** |  | **0.92** |  | **0.92** |  | **7.89** |  | **0.19** |  | **0.001** |
|  |  | Growth stage |  | **2** |  | **0.69** |  | **0.34** |  | **2.68** |  | **0.14** |  | **0.003** |
|  |  | Cultivar |  | 1 |  | 0.16 |  | 0.16 |  | 1.16 |  | 0.03 |  | 0.302 |
