# Supplementary material for: Co-Variation of Bacterial and Fungal Communities in Different Sorghum Cultivars and Growth Stages is Soil Dependent
Source: Microb Ecol. 2017 Nov 16;76(1):205–14. doi: 10.1007/s00248-017-1108-6 (PMC6061463; doi:10.1007/s00248-017-1108-6)
Supplement: Supplementary file 11 — (DOCX 18 kb) [file 248_2017_1108_MOESM11_ESM.docx]

**Table S5**. List of taxonomic groups of bacteria and fungi in rhizosphere that contributed to co-variation in Clue field soil

| **Community** |  | **Taxonomical group** | | | | | | |
| --- | --- | --- | --- | --- | --- | --- | --- | --- |
|  |  | **Phylum** |  | **Class** |  | **Order** |  | **Family** |
| **Bacteria** |  | Bacteroidetes |  | Sphingobacteriia |  | *Sphingobacteriales* |  | *Sphingobacteriaceae* |
|  |  | Proteobacteria |  | Alphaproteobacteria |  | *Rhodospirillales* |  | *Acetobacteraceae* |
|  |  |  |  | Betaproteobacteria |  | *Methylophilales* |  | *Methylophilaceae* |
|  |  |  |  |  |  | *Burkholderiales* |  | *Oxalobacteraceae* |
|  |  |  |  |  |  |  |  | *Alcaligenaceae* |
|  |  | Firmicutes |  | Bacilli |  | *Bacillales* |  | *Planococcaceae* |
|  |  |  |  |  |  | *Lactobacillales* |  | *Lactobacillaceae* |
|  |  |  |  |  |  |  |  |  |
| **Fungi** |  | Ascomycota |  | Dothideomycetes |  | *Capnodiales* |  | unc_Capnodiales |
|  |  |  |  | uncl_Pezizomycotina |  | uncl_Pezizomycotina |  | unc_Pezizomycotina |
|  |  |  |  | Sordariomycetes |  | *Hypocreales* |  | *Nectriaceae* |
|  |  |  |  |  |  |  |  | *Hypocreaceae* |
|  |  | Basidiomycota |  | Tremellomycetes |  | *Tremellales* |  | unc_*Tremellales* |
|  |  | Cryptomycota |  | uncl_LKM11 |  | uncl_LKM11 |  | unc_LKM11 |
|  |  | Glomeromycota |  | Glomeromycetes |  | *Glomerales* |  | unc_*Glomerales* |
