# Supplementary material for: Co-Variation of Bacterial and Fungal Communities in Different Sorghum Cultivars and Growth Stages is Soil Dependent
Source: Microb Ecol. 2017 Nov 16;76(1):205–14. doi: 10.1007/s00248-017-1108-6 (PMC6061463; doi:10.1007/s00248-017-1108-6)
Supplement: Supplementary file 12 — (DOCX 19.1 kb) [file 248_2017_1108_MOESM12_ESM.docx]

**Table S6**. List of taxonomic groups of rhizosphere bacteria and fungi that contributed to co-variation in Vredepeel soil

| **Community** |  | **Taxonomical group** | | | | | | |
| --- | --- | --- | --- | --- | --- | --- | --- | --- |
|  |  | **Phylum** |  | **Class** |  | **Order** |  | **Family** |
| **Bacteria** |  | Acidobacteria |  | Acidobacteria_Gp4 |  | unc_Acidobacteria_Gp4 |  | unc_Acidobacteria_Gp4 |
|  |  |  |  | Acidobacteria_Gp6 |  | unc_Acidobacteria_Gp6 |  | unc_Acidobacteria_Gp6 |
|  |  | Actinobacteria |  | Actinobacteria |  | *Actinomycetales* |  | *Microbacteriaceae* |
|  |  | Bacteroidetes |  | Sphingobacteriia |  | *Sphingobacteriales* |  | *Chitinophagaceae* |
|  |  | Proteobacteria |  | Alphaproteobacteria |  | unc_Alphaproteobacteria |  | unc_Alphaproteobacteria |
|  |  |  |  |  |  | *Rhizobiales* |  | *Bradyrhizobiaceae* |
|  |  |  |  |  |  |  |  | unc_*Rhizobiales* |
|  |  |  |  | Betaproteobacteria |  | *Burkholderiales* |  | *Burkholderiaceae* |
|  |  |  |  | Deltaproteobacteria |  | *Myxococcales* |  | *Polyangiaceae* |
|  |  |  |  |  |  |  |  | unc_*Myxococcales* |
|  |  |  |  | Gammaproteobacteria |  | *Xanthomonadales* |  | *Xanthomonadaceae* |
|  |  |  |  | unc_Proteobacteria |  | unc_Proteobacteria |  | unc_Proteobacteria |
|  |  | Firmicutes |  | Bacilli |  | *Bacillales* |  | *Alicyclobacillaceae* |
|  |  | Verrucomicrobia |  | Opitutae |  | *Opitutales* |  | *Opitutaceae* |
|  |  |  |  |  |  |  |  |  |
| **Fungi** |  | Ascomycota |  | Dothideomycetes |  | *Pleosporales* |  | *Pleosporaceae* |
|  |  |  |  | Saccharomycetes |  | *Saccharomycetales* |  | Saccharomycetales I.S. |
|  |  |  |  | Pezizomycetes |  | *Pezizales* |  | unc_*Pezizales* |
|  |  |  |  | Sordariomycetes |  | *Diaporthales* |  | unc_*Diaporthales* |
|  |  |  |  |  |  | *Hypocreales* |  | unc_*Hypocreales* |
|  |  | Basidiomycota |  | Agaricomycetes |  | *Auriculariales* |  | *Auriculariaceae* |
|  |  | Chytridiomycota |  | Chytridiomycetes |  | *Spizellomycetales* |  | unc_*Spizellomycetales* |
|  |  | Glomeromycota |  | Glomeromycetes |  | *Paraglomerales* |  | *Paraglomeraceae* |
|  |  |  |  |  |  | *Glomerales* |  | *Glomeracea* |
|  |  |  |  |  |  |  |  | unc_*Glomerales* |
|  |  | Zygomycota |  | Mucoromycotina_I.S. |  | *Mucorales* |  | unc_*Mucorales* |
