# Supplementary material for: Co-Variation of Bacterial and Fungal Communities in Different Sorghum Cultivars and Growth Stages is Soil Dependent
Source: Microb Ecol. 2017 Nov 16;76(1):205–14. doi: 10.1007/s00248-017-1108-6 (PMC6061463; doi:10.1007/s00248-017-1108-6)
Supplement: Supplementary file 14 — (DOCX 20 kb) [file 248_2017_1108_MOESM14_ESM.docx]

**Table S8**. Alpha diversity calculation of soil fungi samples comprising estimators of diversity (Shannon), Number of Operation Taxonomic Unites (OTU’s) and estimators of richness (Chao1) given by environmental DNA in two cultivars (BRS330 and SRN-39); in three different plant growth stages (days 10, 35 and 50) in Bulk and in rhizosphere compartments of Clue Field (CF) and Vredepeel (VD) soil

| α-diversity |  | Soil |  | Bulk soil |  | Rhizosphere | | | | | | |
| --- | --- | --- | --- | --- | --- | --- | --- | --- | --- | --- | --- | --- |
| index |  |  |  |  |  | Cultivars |  | Day 10 |  | Day 35 |  | Day 50 |
| Shannon |  |  |  |  |  |  |  |  |  |  |  |  |
|  |  | CF |  | 5.2 ± 0.1 Aa |  | BRS330 |  | 4.2 ± 0.1 Aa |  | 5.1 ± 0.3 Aa |  | 5.5 ± 0.1 Aa |
|  |  |  |  |  |  | SRN-39 |  | 4.2 ± 0.4 Aa |  | 4.9 ± 0.2 Aa |  | 5.0 ± 0.1 Aa |
|  |  |  |  |  |  |  |  |  |  |  |  |  |
|  |  | VD |  | 5.2 ± 0.2 Aa |  | BRS330 |  | 4.4 ± 0.6 Aa |  | 5.3 ± 0.1 Aa |  | 5.5 ± 0.1 Aa |
|  |  |  |  |  |  | SRN-39 |  | 5.2 ± 0.2 Aa |  | 4.9 ± 0.3 Aa |  | 5.4 ± 0 Aa |
| OTUs |  |  |  |  |  |  |  |  |  |  |  |  |
|  |  | CF |  | 96.3 ± 2.8 Aa |  | BRS330 |  | 76.1 ± 3.8 Aa |  | 99.7 ± 8 Aa |  | 101 ± 2.8 Aa |
|  |  |  |  |  |  | SRN-39 |  | 80.1 ± 7.2 Aa |  | 85 ± 6.6 Aa |  | 88.5 ± 3.3 Aa |
|  |  |  |  |  |  |  |  |  |  |  |  |  |
|  |  | VD |  | 90 ± 2.4 Aa |  | BRS330 |  | 75.2 ± 8.3 Aa |  | 87.6 ± 4.9 Aa |  | 100.9 ± 2.1 Aa |
|  |  |  |  |  |  | SRN-39 |  | 91.1 ± 4.3 Aa |  | 83 ± 6.6 Aa |  | 95.3 ± 2.1 Aa |
| Chao1 |  |  |  |  |  |  |  |  |  |  |  |  |
|  |  | CF |  | 146.4 ± 2.3 Aa |  | BRS330 |  | 139.3 ± 14.2 Aa |  | 170.56 ± 17.5 Aa |  | 159.9 ± 1.8 Aa |
|  |  |  |  |  |  | SRN-39 |  | 142 ± 13.2 Aa |  | 139.2 ± 17.7 Aa |  | 148.3 ± 7.6 Aa |
|  |  |  |  |  |  |  |  |  |  |  |  |  |
|  |  | VD |  | 157.8 ± 11.6 Aa |  | BRS330 |  | 119.1 ± 14 Aa |  | 127.3 ± 4.8 Aa |  | 166.2 ± 8.6 Aa |
|  |  |  |  |  |  | SRN-39 |  | 147.9± 3.3 Aa |  | 141.4 ± 7.6 Aa |  | 154.5 ± 8.9 Aa |

The values are means of replicates (n=3) ± (SE). For each α-diversity index capital letters compare (column) the means between the soils within the same soil compartment, cultivar, and time point. Lowercase letters compare (within the same soil), the means either between soil compartments, cultivars within (column) stages of plant growth or the same cultivar (row) over different growth stages. Means followed by the same letter are not statistically different by Tukey test (P<0.05). The sequences were rarefied by 550 reads prior the analysis.
